# Antitumor activity of T cells secreting αCD133-αCD3 bispecific T-cell engager against cholangiocarcinoma

**DOI:** 10.1371/journal.pone.0265773

**Published:** 2022-03-21

**Authors:** Thanich Sangsuwannukul, Kamonlapat Supimon, Thaweesak Chieochansin, Kornkan Choomee, Jatuporn Sujjitjoon, Mutita Junking, Pa-thai Yenchitsomanus

**Affiliations:** 1 Siriraj Center of Research Excellence for Cancer Immunotherapy (SiCORE-CIT), Research Department, Faculty of Medicine Siriraj Hospital, Mahidol University, Bangkok, Thailand; 2 Division of Molecular Medicine, Research Department, Faculty of Medicine Siriraj Hospital, Mahidol University, Bangkok, Thailand; Sapporo Medical University School of Medicine, JAPAN

## Abstract

Cholangiocarcinoma (CCA) is a lethal cancer of bile duct epithelial cells with a high mortality rate and limited therapeutic options. An effective treatment is, therefore, urgently needed to improve treatment outcomes for these patients. To develop a new therapeutic option, we engineered T cells secreting αCD133-αCD3 bispecific T-cell engager and evaluated their antitumor effects against CD133-expressing CCA cells. The cDNA encoding αCD133-αCD3 bispecific T-cell engager (αCD133-αCD3-ENG) was cloned into pCDH lentiviral construct and its expression was tested in Lenti-X 293T cells. T cells from healthy donors were then transduced with engineered lentiviruses to create T cells secreting αCD133-αCD3 engager to evaluate their antitumor activities. The average transduction efficiency into T cells was approximately 60.03±21.65%. In the co-culture system containing T cells secreting αCD133-αCD3 engager (as effector cells) and mWasabi-luciferase-expressing CCA cells (KKU-100 and KKU-213A; as target cells), the effector T cells exhibited significantly higher cytolytic activities against the target CCA cells (49.0±9.76% and 64.10±13.18%, respectively) than those observed against the untransduced T cells (10.97±10.65%; *p* = 0.0103 and 9.80±11.05%; *p* = 0.0054) at an effector-to-target ratio of 5:1. In addition, the secreted αCD133-αCD3 engager significantly redirected both transduced T cells and bystander T cells to kill the target CCA cells (up to 73.20±1.68%; *p*<0.05). Moreover, the transduced and bystander T cells could kill the target CCA spheroids at a rate approximately 5-fold higher than that of the no treatment control condition (*p* = 0.0011). Our findings demonstrate proof-of-principle that T cells secreting αCD133-αCD3 engager can be an alternative approach to treating CD133-positive CCA, and they pave the way for future *in vivo* study and clinical trials.

## Introduction

Cholangiocarcinoma (CCA) is a lethal and heterogeneous biliary tract cancer. Surgery is the only curative treatment, but it can be ineffective in the advanced stages of disease, so novel methods to treat CCA are crucially required [1–3]. The heterogeneity of CCA can be partly explained by the fact that CCA is originated from cancer stem cells (CSC), which is the rationale for targeted therapy specific to surface CSC markers [4–6]. Many potential CSC markers have been reported for CCA treatments, including CD24, CD44, CD90, and CD133 [5]. To target specific tumor-associated antigen (TAA) expressed on tumor cells without MHC restriction, T cell redirecting strategies represent a promising approach for cancer treatment [7]. Chimeric antigen receptor (CAR) T cell therapy is a T cell redirecting strategy that has shown impressive outcomes in cancer treatment [8, 9]. Apart from CAR T cell therapy, modified T cells to secrete bispecific antibody is an attractive approach that can overcome some challenges of passive administration of bispecific antibody [7]. Bispecific T cell engager (commercially called BiTE) is an engineered protein consisting of two single chain variable fragments (scFvs) that are fused to contain bi-functional domains–one targeting TAA expressed on tumor cells, and the other targeting CD3 chains on T cells. Thus, BiTE is able to redirect T cells to tumor cells expressing specific TAA, leading to tumor cell lysis [10, 11]. Blinatumomab (CD19-BiTE) has demonstrated clinical benefit and has been approved by the United States Food and Drug Administration (US FDA) for hematologic malignancies, and other BiTEs have shown potential in different tumor models. However, a lack of biodistribution and a short half-life were their major shortcomings [7, 12, 13]. To overcome these challenges, an alternative method using T cells engineered to secrete BiTE, which represents a combination of antibody-based therapy and T cell-based therapy, is an encouraging approach [7]. The T cells secreting bispecific T cell engager are modified T cells that *in situ* secrete engager molecules to engage themselves and also to recruit bystander T cells to kill cancer cells [14]. Preclinical studies in T cells secreting engager molecules reported effectiveness against different cancer models both *in vitro* and *in vivo* [14–16]. However, data specific to the use of T cells secreting engager molecules in solid tumors, especially CCA, remain scarce.

Although no uniquely specific antigen has been identified in CCA, CD133 is a potential candidate surface antigen for CCA treatment, and has also been defined as a CSC marker in various types of cancers [17–19]. CD133 is one of the most extensively studied CSC markers, and it is a target of CAR T cell therapy that has reached phase I clinical trials [20, 21]. The effectiveness of the CD133 bispecific antibody has been studied in colorectal cancer and glioblastoma stem cells [22, 23]; however, the study of T cells secreting engager molecules in CCA has not yet been reported. In this study, we set forth to create genetically modified T cells secreting αCD133-αCD3 bispecific T cell engager molecules (αCD133-αCD3 ENG) via transduction of T cells isolated from peripheral blood mononuclear cells (PBMCs) with lentiviruses carrying cDNA encoding αCD133-αCD3-ENG, and to evaluate their CCA cell killing efficiency.

## Materials and methods

### Cell lines and cell culture

The human cell lines KKU-100 [24], KKU-213A (cholangiocarcinoma cells) [25] and MMNK-1 (immortal cholangiocytes) [26] were obtained from the Japanese Collection of Research Bioresources (JCRB) Cell Bank (Osaka, Japan). The mWasabi-firefly-luciferase (wLuc) overexpressing KKU-100, KKU-213A and MMNK-1 cells were established and maintained in our laboratory [27, 28]. Lenti-X 293T cell line was purchased from Takara Bio, Inc. (Shiga, Japan) and maintained in 10% heat-activated fetal bovine serum (FBS) (Gibco; Thermo Fisher Scientific, Waltham, MA, USA) supplemented Dulbecco’s modified Eagle’s media (DMEM) at 37°C in a humidified 5% CO_2_ atmosphere.

### Generation of a lentiviral construct containing cDNA encoding αCD133-αCD3 engager

The sequence of codon-optimized cDNA encoding a signal peptide preceding anti-CD133 scFv derived from GenBank number HW350341.1 and anti-CD3 scFv derived from OKT3 [29] with a short serine-glycine linker and myc-tag in tandem was designed and synthesized by Integrated DNA Technologies (Coralville, IA, USA). The synthesized cDNA was subcloned into an in-house pCDH.EF1α.RFP.SIN.WPRE lentiviral vector containing red fluorescent protein (RFP) and T2A using EcoRI/NotI restriction sites to obtain the pCDH.EF1α.RFP.T2A.αCD133-αCD3-ENG.SIN.WPRE (pCDH.αCD133-αCD3-ENG) lentiviral construct. The pCDH.αCD133-αCD3-ENG construct was transformed into *Stbl3* competent *Escherichia coli* and selected by ampicillin. The plasmid construct was isolated and its sequence was confirmed by Sanger DNA sequencing.

### Expression of αCD133-αCD3 engager in Lenti-X 293T cells

#### Analysis by flow cytometry

Lenti-X 293T cells were plated and cultured for 24 hours prior to experiments. The cells were transfected with pCDH.αCD133-αCD3-ENG using Lipofectamine 2000^®^ (Thermo Fisher Scientific). After culturing for 48 hours, the transfected cells were harvested, washed three times with 1x phosphate-buffered saline (PBS) and fixed with 4% paraformaldehyde in 2% FBS in 1x PBS (2% FBS/PBS). Stable Lenti-X 293T cells expressing αCD133-αCD3 engager were generated via lentiviral transduction and selection with 1 μg/mL puromycin. RFP expression was assessed using a FACSVerse^™^ flow cytometer (BD Biosciences, Franklin Lakes, NJ, USA), and analyzed by using FlowJo software (FlowJo LLC, Ashland, OR, USA).

#### Analysis by immunofluorescence staining

Lenti-X 293T cells that stably expressed αCD133-αCD3 engager were plated on coverslips and fixed with 4% paraformaldehyde. The cells were then blocked with 2% FBS in 1x PBS for an hour, and stained with rabbit anti-myc-tag primary antibody (ab9106; Abcam, Cambridge, United Kingdom) at a dilution of 1:500 for 1 hour at room temperature (RT), followed by staining with Alexa Fluor^®^ 488-labeled donkey anti-rabbit secondary antibody for 1 hour with Hoechst 33342 (Abcam) for nuclei counterstaining. After washing three times, coverslips were sealed onto the glass slides. Images were acquired using a Zeiss LSM 800 confocal microscope (Jena, Germany) at the Division of Molecular Medicine, Siriraj, Mahidol University.

#### Analysis by immunoblotting assay

For immunoblotting assay, the Lenti-X 293T cells stably expressing αCD133-αCD3 engager molecules and their culture supernatant were harvested. Whole cell lysates were prepared using radioimmunoprecipitation assay (RIPA) lysis buffer on ice for 30 minutes with subsequent centrifugation at 4°C and 13,000 revolutions per minute (rpm) for 30 minutes. The proteins were resolved on 12% sodium dodecyl sulfate-polyacrylamide gel electrophoresis (SDS-PAGE) and then transferred onto a sheet of nitrocellulose membrane. The proteins on the membrane were probed with mouse-anti-myc primary antibody (9E10; Santa Cruz Biotechnology, Dallas, TX, USA) and rabbit anti-mouse HRP-conjugated secondary antibody.

### Peripheral blood mononuclear cell (PBMC) preparation and lymphocyte isolation

The protocol for the human sample study was approved by the Siriraj Institutional Review Board (SIRB) of the Faculty of Medicine Siriraj Hospital, Mahidol University, Bangkok, Thailand (COA no. *Si* 101/2020). All healthy donors provided written informed consent before blood sample collection. All procedures in this study were conducted in compliance with the Declaration of Helsinki and ICH Good Clinical Practice Guidelines. Human peripheral blood mononuclear cells (PBMCs) were isolated from blood samples obtained from healthy donors using lymphocyte separation medium (Corning, Inc., Corning, NY, USA) and gradient centrifugation. Lymphocytes were separated and stimulated (day 0) as previously described [27]. For functional study, the cells were transduced with engineered lentiviruses on day 3 after stimulation and maintained in medium containing cytokines (see below).

### Viral packaging and T cell transduction

Lentiviral particles containing the αCD133-αCD3-ENG sequence were packaged in Lenti-X 293T cells (Takara Bio) by co-transfection of the pCDH.αCD133-αCD3-ENG plasmid with the packaging plasmids psPAX2 and pMD2.G at a ratio of 21:6:2 μg. Concentrated lentivirus was obtained using a Sorvall RC-6 Plus centrifuge (Thermo Fisher Scientific) at 20,000 g and 4°C for 150 minutes. The lentiviruses at a multiplicity of infection (MOI) of 10 each were used for double transduction of activated T cells on days 3 and 5. The cells were then cultured in medium containing human IL-2, IL-7, and IL-15 cytokines (Immunotools, Friesoythe, Germany) for 5–7 days, and the transduction efficiency of the T cells was determined by FACSVerse^TM^ flow cytometer (BD Biosciences).

### Analysis of T cell immunophenotypes

Anti-CD3-FITC (Clone UCHT-1), anti-CD4-APC (Clone MEM-241), anti-CD8-APC (Clone UCHT-4), and anti-CD62L-APC (Clone LT-TD180) were purchased from Immunotools. Anti-CD45RA-PE-cy7 (Clone HI100) was purchased from Invitrogen (Carlsbad, CA, USA). Freshly isolated PBMCs (1×10^6^ cells) and the transduced T cells (1×10^5^ cells) were incubated with specific antibodies (1:100) at 4°C for 45 minutes, and then examined by FACSVerse™ flow cytometer (BD Biosciences). The acquired data was analyzed using FlowJo software.

### Cytolytic assay of bystander T cells against CCA cells

To examine the effect of αCD133-αCD3 engager molecules secreted from the transduced T cells to redirect bystander T cells to kill target CCA cells, 5×10^4^ wLuc-KKU-213A cells expressing the CD133 antigen were placed into the lower chamber of a well of a 24-well plate. Activated (untransduced) T cells (ATCs) at 2.5×10^5^ cells were added into the same well. A 0.4 μm pore Transwell insert (Corning, NY USA) was then placed into the well. After that, untransduced T cells or transduced T cells (at a ratio of 5:1) were added into the upper chamber. After co-culturing for 72 hours, the viable tumor cells were observed under a fluorescent microscope. The percentage of cytolysis was measured and calculated from luciferase activity using a Pierce™ Firefly Luciferase Glow Assay Kit (Thermo Fisher Scientific), as follows:

$$\%\;Specific\;cytolysis=100\times \frac{\left(Experimental\;data-Spontaneous\;cell\;death\right)}{(Maximum\;cell\;death-Spontaneous\;cell\;death)\;}.$$

### Cytolytic activity assay

The effector T cells and cancer cells were co-cultured at an effector-to-target (E:T) ratio of 1:5 for 3 and 7 days. The cell numbers of effector T cells were determined using a Countess™ II cell counter (Thermo Fisher Scientific). For cytolytic activity assay, 1×10^4^ cells of wLuc-KKU-213A or wLuc-MMNK-1 were co-cultured with either untransduced T cells or T cells secreting αCD133-αCD3 engager at E:T ratios of 1:1, 2.5:1, and 5:1 in a 96-well plate for 18 hours. The percentage of specific cytolysis was measured and calculated from luciferase activity using a Pierce™ Firefly Luciferase Glow Assay Kit (Thermo Fisher Scientific) as described above.

### Cytokine assay

The untransduced T cells or T cells secreting αCD133-αCD3 engager molecules were co-cultured with 2.5×10^4^ KKU-213A cells at an E:T ratio of 5:1 in a 96-well plate. After 24 hours of co-culturing, IFN-ɣ was determined using an enzyme-linked immunosorbent assay (ELISA) kit (Thermo Fisher Scientific) according to the manufacturer’s instructions. Briefly, after co-culturing, the culture supernatant was collected and centrifuged at 2,000 rpm and 4°C for 5 minutes. The samples were then added into assay plates, followed by the addition of washing buffer, antibody conjugate, substrate, and stop solutions. Absorbance at 450 nm was measured using a Synergy™ Mx Microplate Reader (BioTek Instruments, Inc., Winooski, VT, USA), and the data were analyzed. Intracellular cytokine staining of TNF-α and granzyme B was performed after 6 hours of co-culture at E:T ratio of 5:1 in the presence of Brefeldin A (eBioscience, San Diego, CA, USA). Effector cells were harvested and stained with fluorescence-conjugated anti-human CD3 (Clone UCHT-1), followed by staining with anti-human TNF-α (Clone IT-5H2) or anti-human granzyme B (HC4) antibody (Immunotools), in the presence of 0.5% saponin (Sigma-Aldrich) for 45 minutes. The samples were run immediately using flow cytometry.

### Tumor spheroid lysis assay

The tumor spheroid of wLuc-KKU-213A cells was prepared. Briefly, 2×10^3^ tumor cells were mixed with Corning^®^ Matrigel Basement Membrane Matrix (Corning) on ice, seeded into a 96-well clear round bottom ultra-low attachment microplate (Corning), and allowed to grow and form tumor spheroids for 3 days prior to the experiments. The untransduced T cells or T cells secreting αCD133-αCD3 engager at an E:T ratio of 10:1 were added into the tumor spheroids. After co-culturing for 5 days, tumor spheroid images were captured using a Ti-S Intensilight Ri1 NIS-D inverted fluorescent microscope (Nikon Instruments, Inc., Tokyo, Japan). The level of fluorescence was quantified using ImageJ program (National Institutes of Health, Bethesda, MD, USA), and corrected total cell fluorescence (CTCF) was calculated (CTCF = Intergrated density–(Area of selected cell × Mean fluorescence of background readings).

### Data and statistical analyses

All experiments were performed in triplicate. The measured data are presented as mean ± standard deviation (SD). Comparisons of data were performed using Student’s *t*-test, and a *p*-value less than 0.05 was considered to be statistically significant. All statistical tests were performed using GraphPad Prism 7.0 (GraphPad Software, Inc., San Diego, CA, USA).

## Results

### Construction of pCDH.αCD133-αCD3-ENG and expression of αCD133-αCD3 engager protein in Lenti-X 293T cells

**Fig 1:**
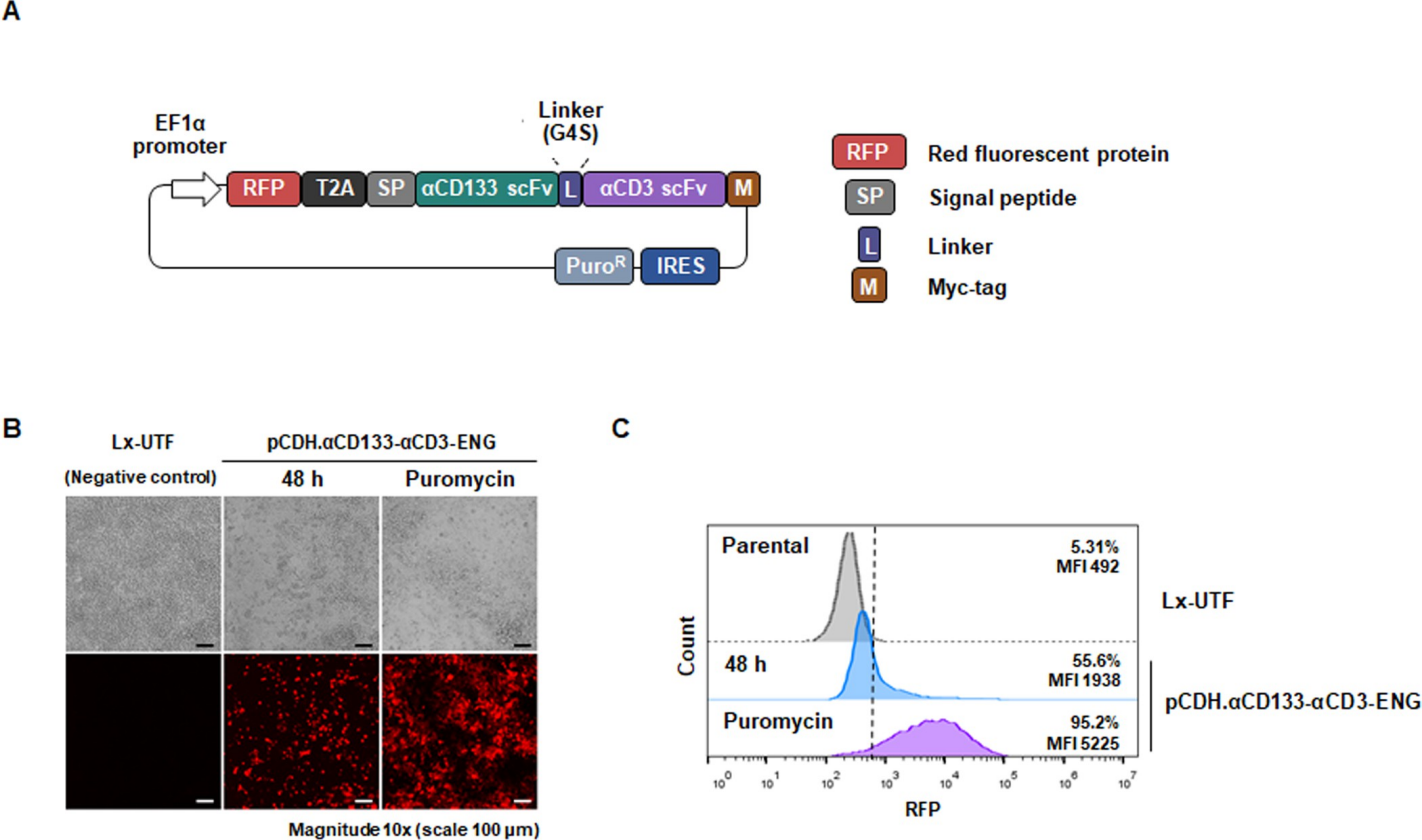
Construction of pCDH.αCD133-αCD3-ENG and expression of αCD133-αCD3 engager protein in Lenti-X 293T cells. (A) Schematic representation of the lentiviral plasmid construct encoding red fluorescence protein (RFP) and αCD133-αCD3 engager with myc-tag. (B) RFP expression in transfected and puromycin selected Lenti-X 293T cells as determined by fluorescence microscopy, and compared to untransfected cells (Lx-UTF). (C) RFP expression in transfected cells before and after puromycin selection as examined by flow cytometry. Scale bar: 100 μm.

To create T cells secreting αCD133-αCD3 engager specific to the CD133 antigen on tumor cells and CD3 on T cells, we first created a lentiviral plasmid construct containing cDNA encoding the αCD133-αCD3 engager protein (pCDH.αCD133-αCD3-ENG) by restriction endonuclease cutting and cloning. The map of the lentiviral transfer plasmid (pCDH.αCD133-αCD3-ENG) consisted of cDNAs encoding RFP, anti-CD133 scFv, and anti-CD3 scFv (Fig 1A). To examine the expression of αCD133-αCD3 engager protein, the lentiviral transfer plasmid (pCDH.αCD133-αCD3-ENG) was transferred into Lenti-X 293T cells. RFP expression was determined by a fluorescence microscope and a flow cytometer using untransfected cells as negative controls. Stable expression of αCD133-αCD3 engager protein in Lenti-X 293T cells was achieved after puromycin selection, which was found to be more than 90% compared to 55.6% among the transiently transfected cells (Fig 1B and 1C).

### Expression and secretion of αCD133-αCD3 engager protein

**Fig 2:**
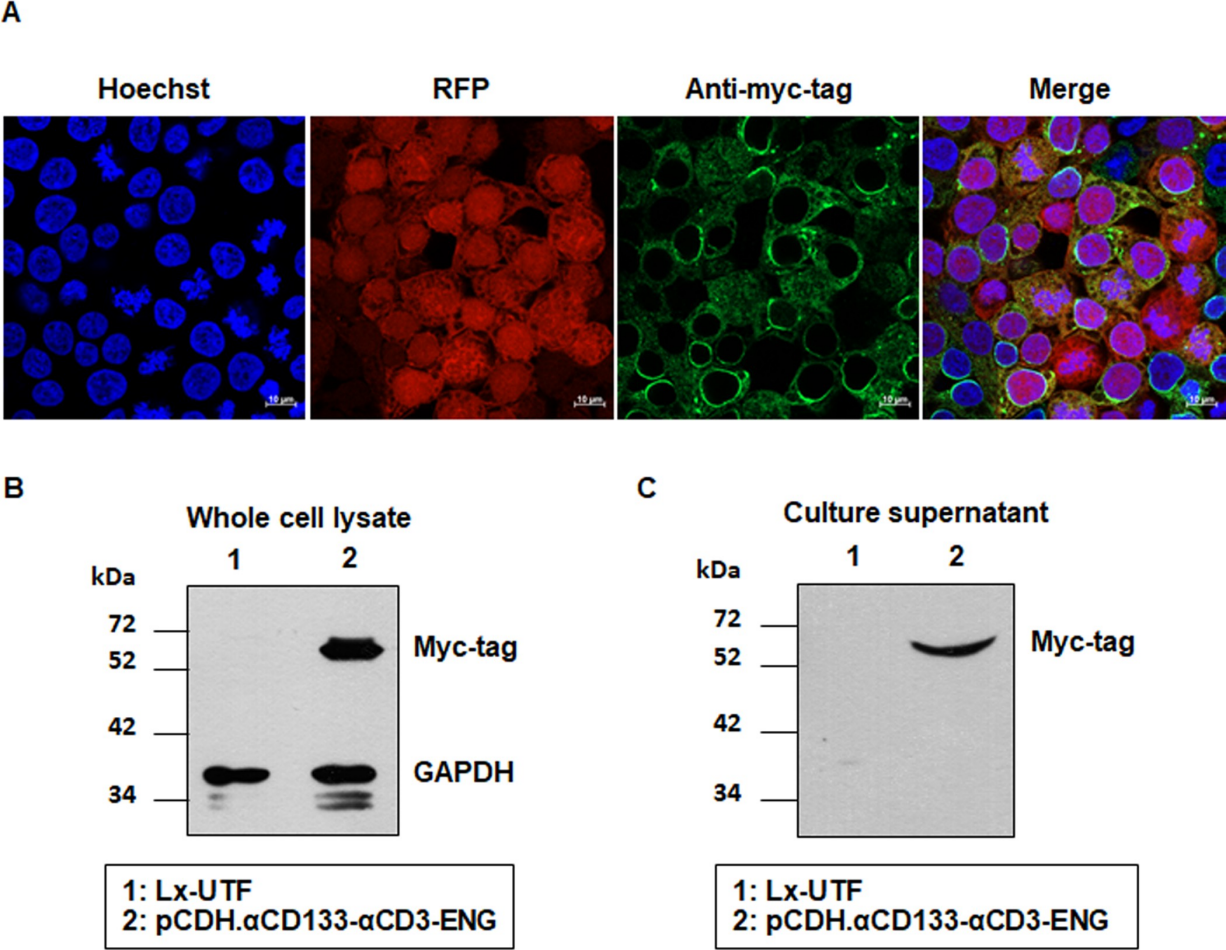
Expression and secretion of the αCD133-αCD3 engager protein. Lenti-X 293T cells were transfected with pCDH.αCD133-αCD3-ENG and selected with puromycin. Intracellular and secreted αCD133-αCD3 engager proteins were examined. (A) Immunofluorescence assay showed co-expression of RFP and myc-tagged αCD133-αCD3 engager proteins in the transfected Lenti-X 293T cells as observed by confocal microscopy. (B) A representative immunoblotting analysis of the αCD133-αCD3 engager protein in the whole cell lysates of transfected and untransfected Lenti-X 293T cells probed with anti-myc and anti-GAPDH monoclonal antibodies. (C) A representative immunoblotting analysis showed the αCD133-αCD3 engager protein in the culture supernatants of the untransfected and transfected Lenti-X 293T cells. Scale bar: 10 μm.

Lenti-X 293T cells were transfected with pCDH.αCD133-αCD3-ENG, and then the intracellular protein expression and secretion were examined. Co-expression of RFP and myc-tagged αCD133-αCD3 engager protein was observed in the transfected Lenti-X 293T cells stably expressing αCD133-αCD3 engager protein (Fig 2A). A whole cell lysate sample of the transfected Lenti-X 293T cells stably expressing αCD133-αCD3 engager protein, and soluble αCD133-αCD3 engager protein secreted in cultured supernatant were examined by immunoblotting assay. The myc-tagged αCD133-αCD3 engager protein was found at a size of approximately 55 kDa in both the whole cell lysate and the culture supernatant (Fig 2B, 2C, and S1 Fig). A significantly high level of myc-tagged αCD133-αCD3 engager protein (104.3±13.34%) was observed in the transfected Lenti-X 293T cells stably expressing the engager protein when compared to that observed in untransfected control cells (7.66±2.21%; *p* = 0.0002) (S1 Fig). The secreted soluble αCD133-αCD3 engager protein was detected in the culture supernatant of the transfected Lenti-X 293T cells stably expressing the engager protein, but it was not detected in the culture supernatant of the parental Lenti-X 293T cells (Fig 2C). These results demonstrated that the generated lentiviral plasmid (pCDH.αCD133-αCD3-ENG) could express αCD133-αCD3 engager protein in Lenti-X 293T cells, and that the engager protein was able to secrete into the culture supernatant.

### Generation of T cells secreting αCD133-αCD3 engager, and characterization of immunophenotypes

**Fig 3:**
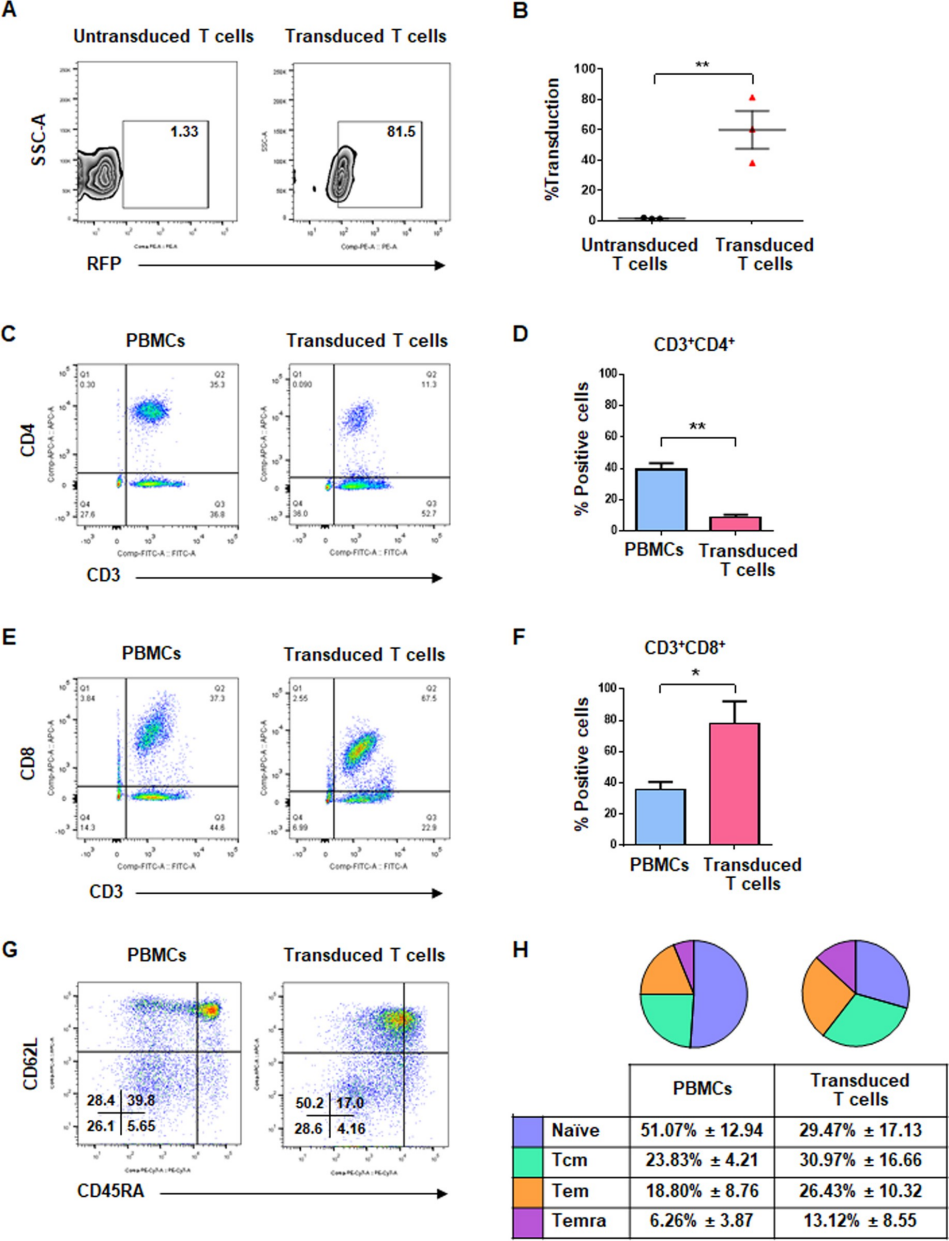
Transduction efficiency, immunophenotypes, and memory subtypes of T cells secreting αCD133-αCD3 engager. (A) Representative data of transduction efficiency and (B) summary data from 3 different healthy donors. (C-F) Immunophenotypes of peripheral blood mononuclear cells (PBMCs) and transdued T cells secreting αCD133-αCD3 engager: (C) representative and (D) summarized data of helper T cells (CD3^+^CD4^+^), and (E) representative and (F) summarized data of cytotoxic T cells (CD3^+^CD8^+^). (G-H) Memory subtypes of PBMCs and transduced T cells secreting αCD133-αCD3 engager: (G) representative and (H) summarized data of three donors. (*p<0.05, **p<0.01).

Lentiviruses carrying the αCD133-αCD3-ENG construct were produced in Lenti-X 293T cells, and their titres of approximately 4.34±3.98⊆10^9^ TU/mL were obtained. The viruses were transduced into T cells isolated from PBMCs to generate T cells secreting αCD133-αCD3 engager. The transduction efficiency ranged from 38.2–81.5% (60.03±21.65%) as determined by RFP expression using flow cytometry (Fig 3A and 3B). After expansion in medium containing cytokines, the immunophenotypes of T cells were characterized. In PBMCs, the percentage of helper T cells (CD3^+^CD4^+^) and cytotoxic T cells (CD3^+^CD8^+^) was 39.40±3.95% and 35.80±5.02%, respectively. In transduced T cells secreting αCD133-αCD3 engager, the percentage of helper T cells (CD3^+^CD4^+^) and cytotoxic T cells (CD3^+^CD8^+^) was 8.71±1.72% (*p* = 0.002) and 78.00±14.20% (*p* = 0.015), respectively (Fig 3C and 3D). Furthermore, our analysis of memory subtypes of T cells showed that in PBMCs, the proportion of naïve, central memory (Tcm), effector memory (Tem), and terminal differentiated effector (Temra) T cell subtypes was 51.07±12.94%, 23.83±4,21%, 18.80±8.76%, and 6.26±3.87%, respectively, whereas in the transduced T cells secreting αCD133-αCD3 engager, they were 29.47±17.13%, 30.97±16.66%, 26.43±10.32%, and 13.12±8.55%, respectively (Fig 3G and 3H). These results demonstrated that the transduced T cells secreting αCD133-αCD3 engager were predominantly cytotoxic T cells (CD3^+^CD8^+^) containing mainly central memory (Tcm) and effector memory (Tem) phenotypes.

### Binding of αCD133-αCD3 engager molecules to untransduced and transduced T cells, and killing of CCA cells by bystander T cells

**Fig 4:**
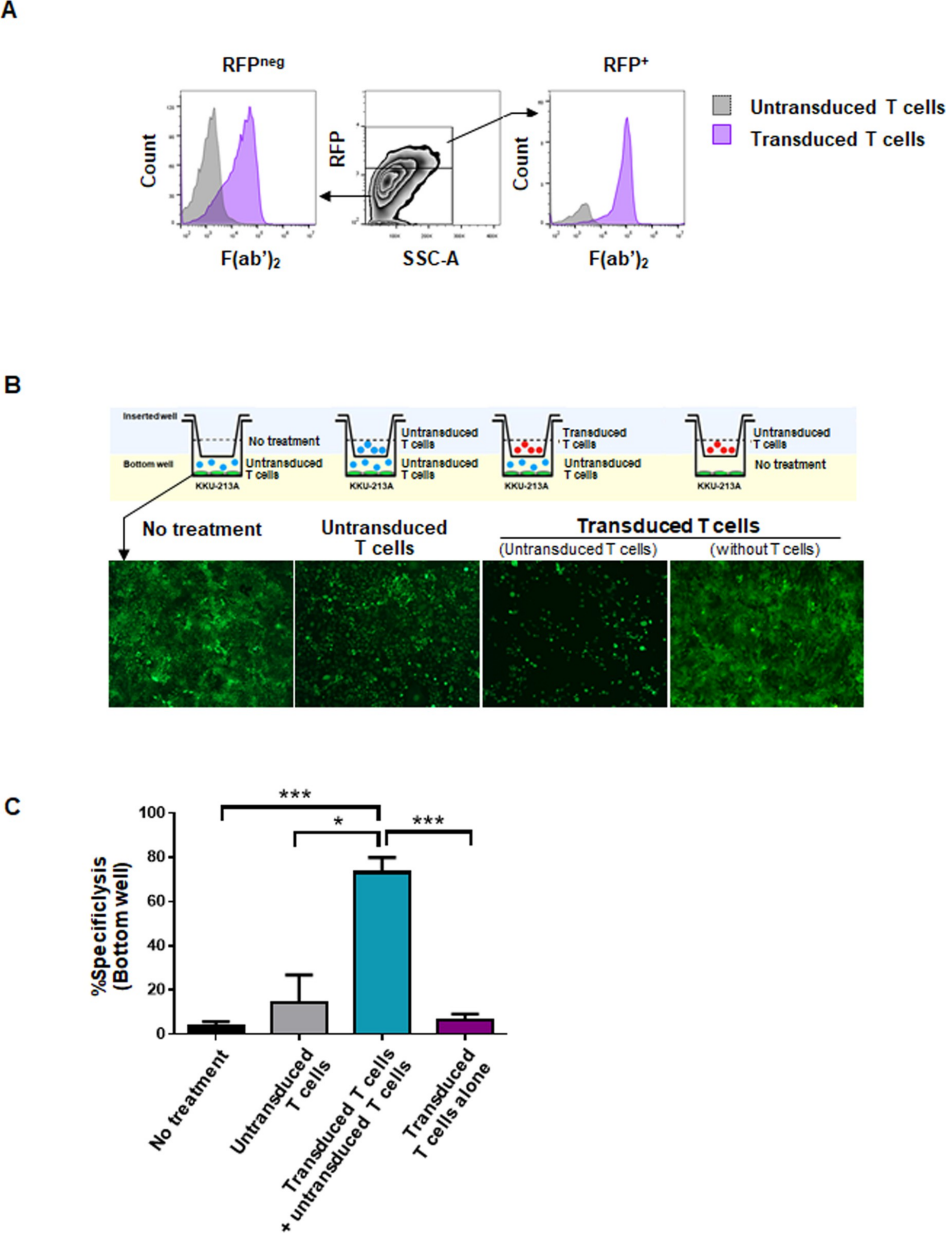
Binding of αCD133-αCD3 engager molecules and killing of CCA cells by bystander T cells. (A) Cell surface binding ability of αCD133-αCD3 engager molecules to the effector T cells; red fluorescent protein (RFP)-negative (untransduced) T cells (left panel) and RFP-positive (transduced) T cells (right panel). (B) Schematic drawing of co-culture in transwell assay (upper panel) and representative microscopic images of wLuc-KKU-213A cells (green) in lower chamber of transwell system after 72 hours of co-culture (lower panel) are shown. (C) Bar graph showing percentages of specific cytolysis of target CCA cells in the lower chamber calculated from luciferase activity of wLuc-KKU-213A cells as described in the text. (*p<0.05, ***p<0.001).

Initially, binding of the secreted αCD133-αCD3 engager molecules to the surface of either untransduced or transduced T cells was investigated by flow cytometry. The presence of F(ab’)_2_ on the surface of both RFP-negative (untransduced) and RFP-positive (transduced) T cells indicated the binding of αCD133-αCD3 engager to both T cell populations (Fig 4A). Next, the ability of αCD133-αCD3 engager to redirect the untransduced or bystander T cells to kill tumor cells was studied using the Transwell system as depicted in Fig 4B. CD133-positive CCA (KKU-213A) cells stably expressing mWasabi-luciferase that were used as target cells were placed into the lower chamber of the wells. Activated (untransduced) T cells were also added with the target CCA cells. The upper chambers were added with medium without T cells, untransduced T cells, or transduced T cells. The results showed that only in the well added with transduced T cells secreting αCD133-αCD3 engager in the upper chambers could the activated (untransduced or bystander) T cells in the lower chamber kill the target CCA cells (Fig 4B) with a significantly higher killing efficiency of 73.2±11.68% (*p*<0.001 when compared to that of no cell addition control, and *p* = 0.015 when compared to that of untransduced T cells). Very low killing effects were observed in the well without addition of T cells in the upper chamber (3.98±3.08%), and in the well with addition of untransduced T cells in the upper chamber (14.25±21.9%). No antitumor effect was observed when transduced T cells were added in the upper chamber, and medium without T cells (target cells alone) were added in the lower chamber (6.42±3.08%) (Fig 4C). These results demonstrated that the αCD133-αCD3 engager could redirect the bystander T cells to kill the target CCA cells.

### Cytotoxic effect, T cell proliferation and IFN-γ cytokine production of transduced T cells secreting αCD133-αCD3 engager against CCA cells

**Fig 5:**
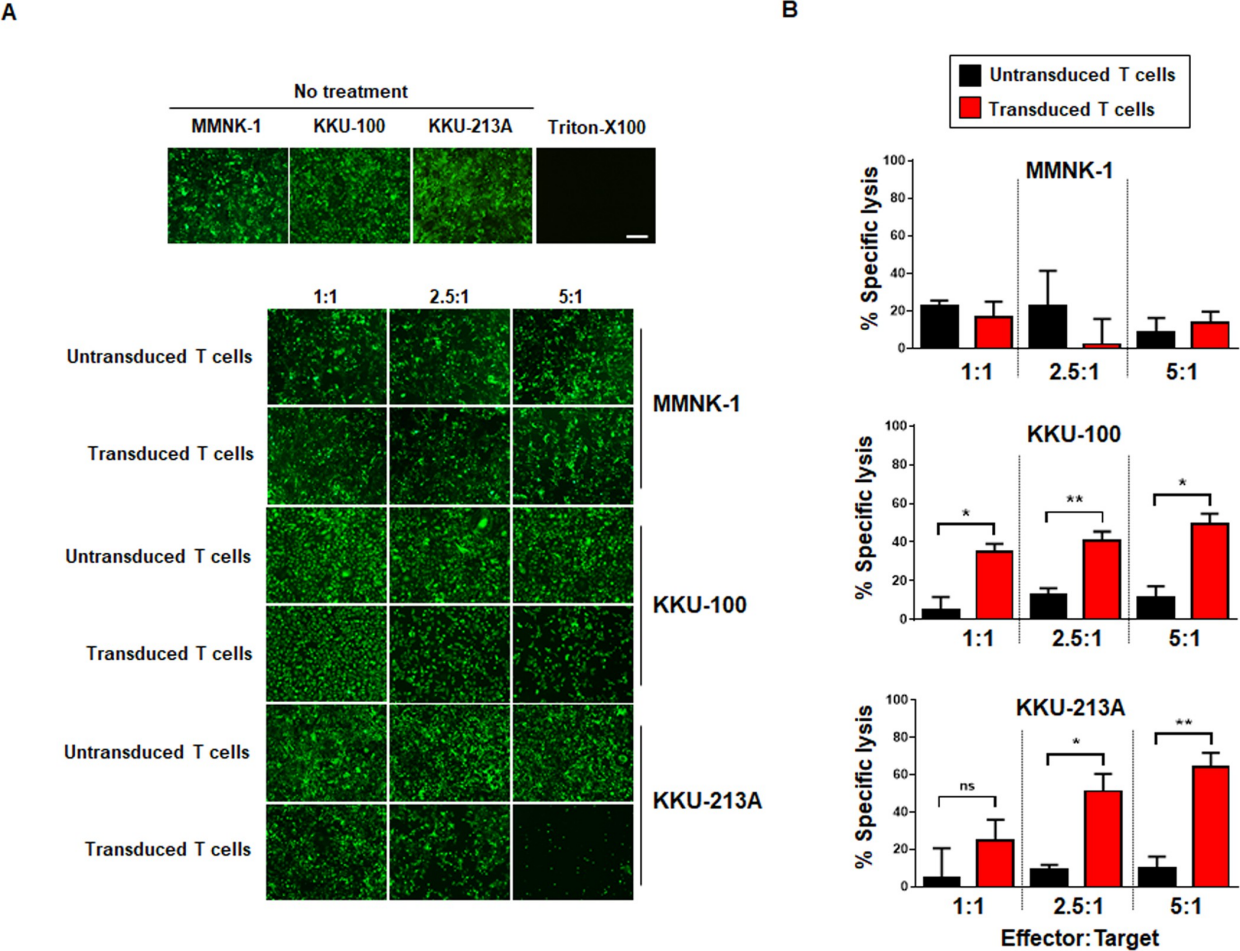
Cytotoxic effect of T cells secreting αCD133-αCD3 engager. (A) The cytotoxic effects of the untransduced and transduced T cells secreting αCD133-αCD3 engager co-cultured with cholangiocytes (MMNK-1) expressing mWasabi-luciferase (wLuc), and of the untransduced and transduced T cells secreting αCD133-αCD3 engager co-cultured with CCA cells expressing both CD133 (KKU-100 and KKU-213A) and mWasabi-luciferase (wLuc). Fluorescent images of viable MMNK-1 and KKU-213A cells (green) were captured after 18 hours of co-culture. (B) The histograms show the percentages of cytotoxic effects calculated from the results of (A). (*p<0.05, **p<0.01, ***p<0.001, NS–not signficantly different).

The surface expression of CD133 in CCA cells was examined by flow cytometry (S2 Fig) [27]; the CD133-expressing KKU-100 and KKU-213A cells served as tumor targets for T cells secreting αCD133-αCD3 engager, compared to low CD133-expressing MMNK-1 cholangiocytes. Antitumor activity of T cells secreting αCD133-αCD3 engager against CD133-expressing CCA cells was examined. The untransduced and transduced T cells secreting αCD133-αCD3 engager showed low cytotoxic effect when they were co-cultured with cholangiocytes (MMNK-1) expressing mWasabi-luciferase (wLuc). While the untransduced T cells exhibited low cytotoxic effect, the transduced T cells secreting αCD133-αCD3 engager demonstrated specific killing activity when they were co-cultured with wLuc-CCA cells expressing CD133 (KKU-100, KKU-213A). Significantly higher cytolytic effects were found when T cells secreting αCD133-αCD3 engager were co-cultured with KKU-100 (*p*<0.05), compared with the results of the untransduced T cell treatments. The specific killing of T cells secreting αCD133-αCD3 engager against KKU-100 at the effector-to-target (E:T) ratios of 1:1, 2.5:1 and 5:1 were 34.88±7.11%, 40.55±8.42%, and 49.00±9.76%, respectively. Similar results were observed in the study using KKU-213A cells. The highest cytotoxic effect of the T cells secreting αCD133-αCD3 engager against KKU-213A cells at an E:T ratio of 5:1 was 64.10±13.18%, compared to the untransduced T cells at the same E:T ratio, which was 9.79±11.05% (*p* = 0.005) (Fig 5).

**Fig 6:**
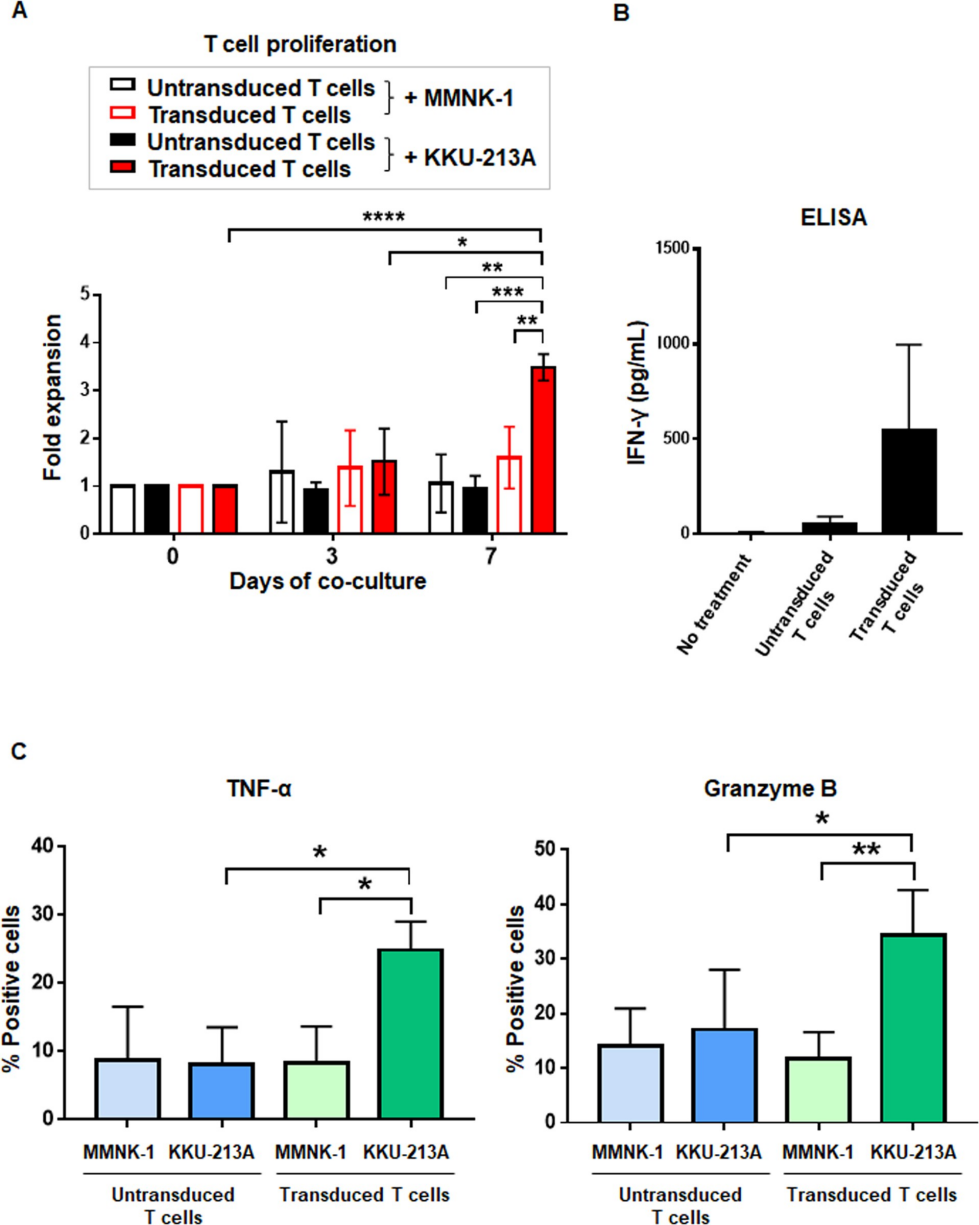
Proliferation and cytokine production of T cells secreting αCD133-αCD3 engager. (A) Proliferation of the untransduced or transduced T cells secreting αCD133-αCD3 engager after being co-cultured with cholangiocytes (MMNK-1) and CD133^+^ KKU-213A cells for 0, 3, and 7 days. (B) Levels of IFN-γ production in culture medium after the untransduced or transduced T cells secreting αCD133-αCD3 engager were co-cultured with CD133^+^ KKU-213A cells for 18 hours as determined by enzyme-linked immunosorbent assay (ELISA). (C) TNF-α and granzyme B intracellular cytokine staining of the untransduced and transduced T cells secreting αCD133-αCD3 engager co-cultured with either cholangiocytes (MMNK-1) or CD133-expressing KKU-213A cells after 6 hours of the co-culture. (*p<0.05, **p<0.01, ***p<0.001).

Then, T cell proliferation and cytokine production were examined by co-culturing of untransduced T cells or transduced T cells secreting αCD133-αCD3 engager with either CD133 expressing KKU-213A or MMNK-1 cholangiocytes for 0, 3, and 7 days. The untransduced or transduced T cells did not proliferate when they were co-cultured with cholangiocytes (MMNK-1 cells). Similarly, the untransduced T cells did not proliferate when they were co-cultured with KKU-213A (Fig 6A). However, the transduced T cells secreting αCD133-αCD3 engager significantly proliferated when they were co-cultured with KKU-213A for 7 days (Fig 6A). IFN-γ cytokine production was increased when the transduced T cells secreting αCD133-αCD3 engager were co-cultured with CCA expressing CD133 (KKU-213A) (Fig 6B). In addition, significant upregulations of TNF-α (*p* = 0.014) and granzyme B (*p* = 0.003) were found when the transduce T cells secreting αCD133-αCD3 engager were co-cultured with KKU-213A, but did not when they were co-cultured with MMNK-1 cells. Moreover, no upregulation of TNF-α and granzyme B was observed when the transduce T cells secreting αCD133-αCD3 engager were co-cultured with the untransduced T cells (Fig 6C).

### Cytotoxic effects of untransduced and transduced T cells secreting αCD133-αCD3 engager against CCA spheroids

**Fig 7:**
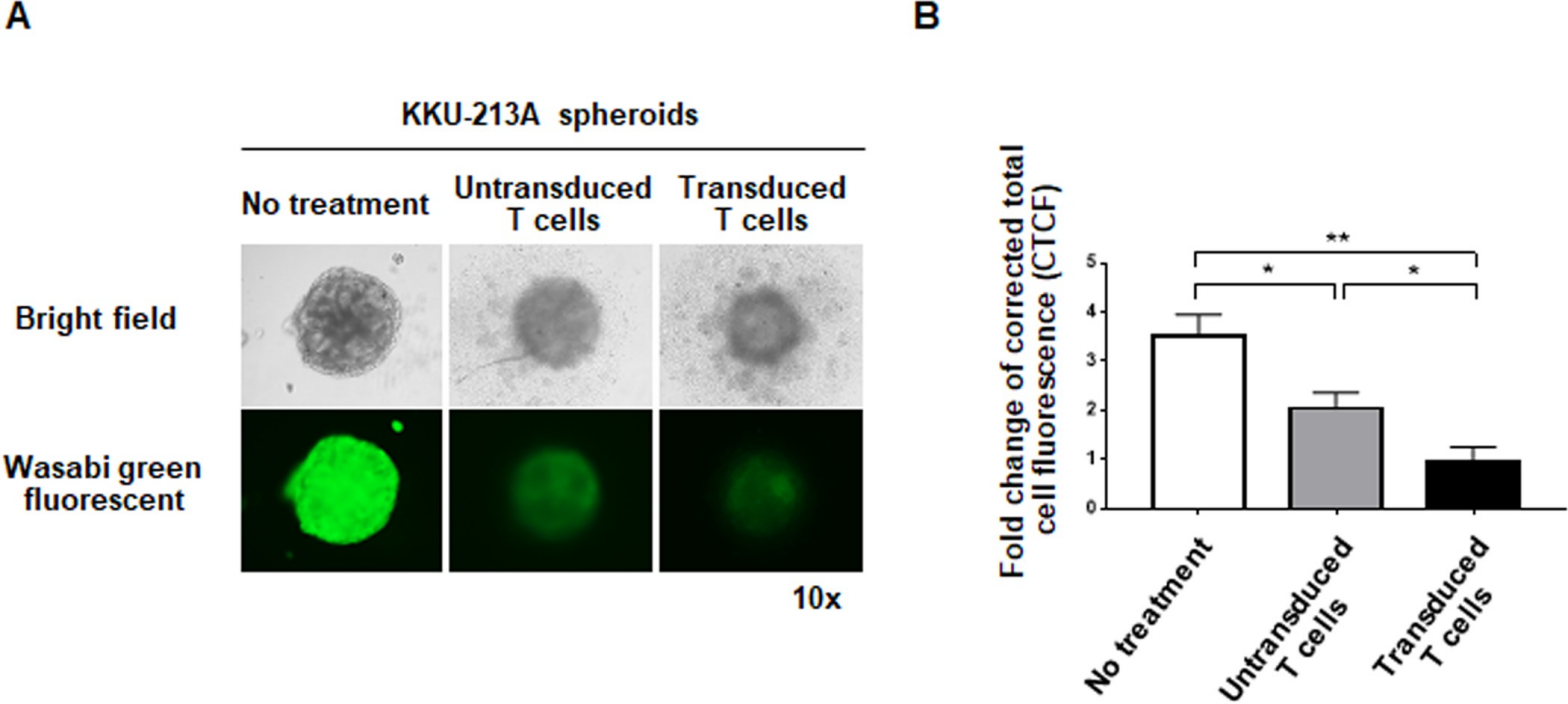
The cytotoxic effects of untransduced and transduced T cells secreting αCD133-αCD3 engager against CCA spheroids. (A) Representative images of CCA spheroids of wLuc-KKU-213A cells in the following conditions: without treatment, co-cultured with untransduced T cells, and co-cultured with transduced T cells secreting αCD133-αCD3 engager. (B) Histograms of summarized green fluorescent signal data from three independent experiments. (*p<0.05, **p<0.01).

To conduct an experiment using three-dimensional (3D) structure of target CCA cells to mimic the tumor mass, CCA spheroids of wLuc-KKU-213A cells were created [28] and co-cultured with the untransduced and transduced T cells secreting αCD133-αCD3 engager. After co-culturing for 5 days, the CCA spheroids were observed under a fluorescence microscope. The results showed that while the shape and green fluorescent signal of the CCA spheroid without treatment were still intact and bright, the shapes and green fluorescent signals of the CCA spheroids co-cultured with the transduced T cells secreting αCD133-αCD3 engager were disrupted and markedly reduced (Fig 7A). The average green fluorescent signals (n = 3 each) of the CCA spheroids co-cultured with untransduced T cells or transduced T cells secreting αCD133-αCD3 engager were significantly reduced [1.89-fold (*p* = 0.045), and 5.67-fold (*p* = 0.001), respectively] compared to the no treatment control (Fig 7B). A greater reduction in green fluorescent signal (2.99-fold) was observed when the CCA spheroid was co-cultured with the transduced T cells secreting αCD133-αCD3 engager (*p* = 0.045), which indicated a more pronounced cytotoxic effect compared to that observed after co-culturing with the untransduced T cells.

## Discussion and conclusion

Immunotherapies targeting CD133 by bispecific antibody (bsAb) and adoptive T cell therapy (ACT) are potential treatments for several cancers, including colorectal cancer, glioblastoma, and cholangiocarcinoma [20, 22, 23, 27, 30]. These antigen-specific immunotherapies can overcome the problem of immune evasion by downregulation of MHC molecules on the cancer cell surface since the immune cells are redirected to eliminate the cancer cells without the requirement of MHC-dependent antigen presentation [7, 31]. CAR T cells and BiTEs are the two T cell redirection strategies that have demonstrated remarkable success in the treatment of CD19-positive cancers [32, 33]. Despite the fact that recombinant BiTEs have potent anti-cancer effects and can redirect T cells to destroy cancer cells, they require long-term infusion and their bio-distributions are limited [7, 34]. Thus, an alternative platform using engineered T cells to secret bispecific T cell engager (ENG) molecules and to engage T cells to aggress against cancer cells is a promising approach for cancer treatment [14–16]. However, this new platform is in the process of development and is being evaluated in several cancer models, including CCA.

Since a uniquely specific antigen is not present in CCA cells, CD133 –a potential cancer stem cell marker [19]–was selected as a target molecule in the T cells that we developed to secrete engager molecules, We selected CD133 based on the following reported evidence: CD133 was shown to be effectively targeted by CAR T cells [20, 27, 35]; CD133 was found to be highly expressed in patient CCA tissue samples [36, 37]; and, CD133 was reported to be associated with reduced rates of cancer relapse and decreased resistance to cancer treatments [6, 18]. We, therefore, set forth to create T cells secreting αCD133-αCD3 bispecific T cell engager (αCD133-αCD3 engager) to engage and activate T cells against CCA cells, and to evaluate its anti-cancer effects in CD133-positive CCA cells and spheroids.

**Fig 8:**
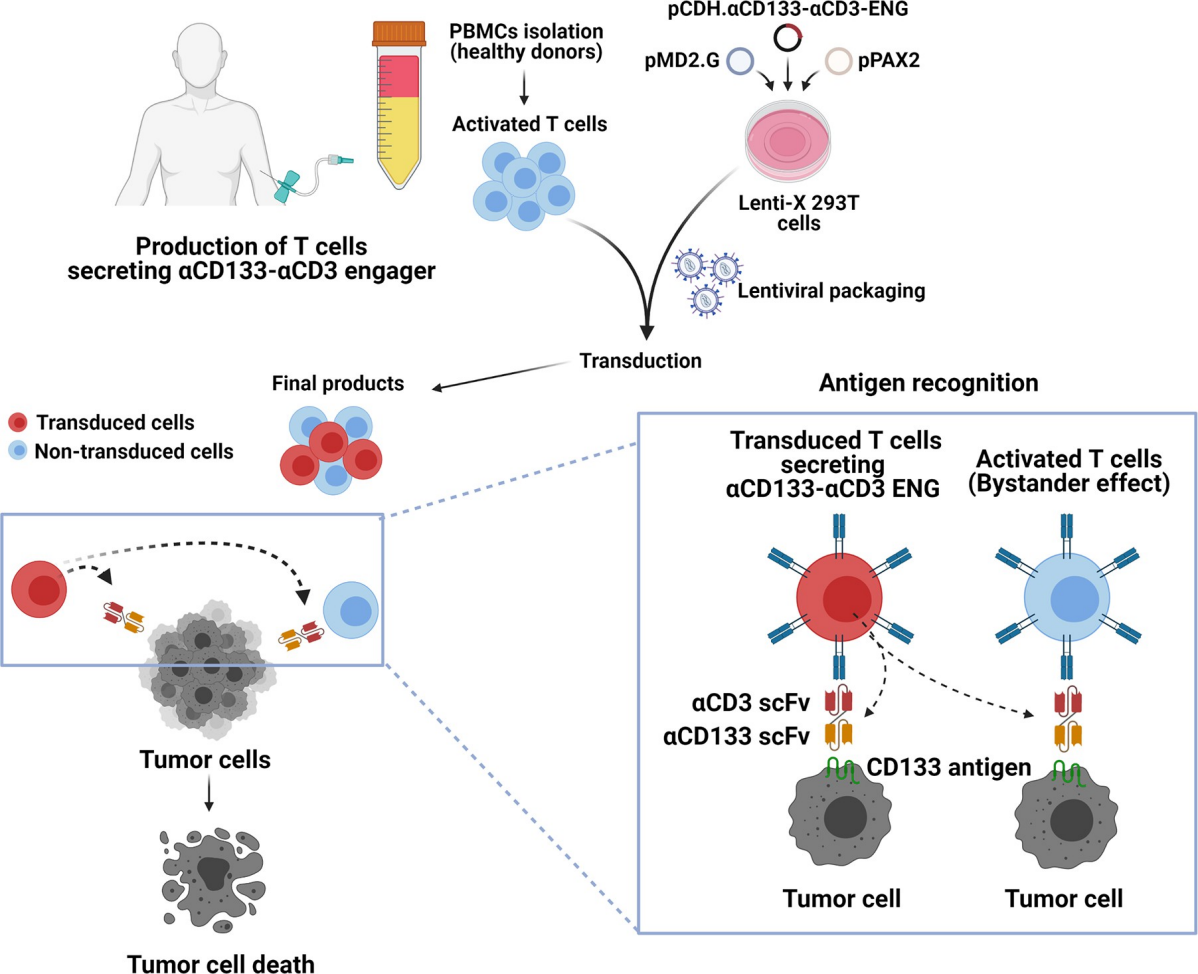
Protocol for the concept and development of T cells secreting αCD133-αCD3 bispecific T cell engager (αCD133-αCD3 ENG). (A) Production of T cells secreting αCD133-αCD3 engager. Peripheral blood mononuclear cells (PBMCs) were isolated and transduced with lentiviruses carrying cDNA encoding αCD133-αCD3-ENG to obtain final products, which are a mixture of untransduced and transduced T cells. (B) The αCD133-αCD3 engager molecules are secreted from the transduced T cells, which can recognize and bind to CD133 tumor-associated antigen expressed on tumor cells. (C) The inset shows the binding of αCD133-αCD3 engager molecules to transduced T cells and untransduced (bystander) T cells, which activates T cells to kill cancer cells. (Image created with BioRender.com).

The overall concept and procedure of the development of T cells secreting αCD133-αCD3 engager are summarized in Fig 8. Initially, a lentiviral construct (pCDH.αCD133-αCD3-ENG) carrying cDNA encoding anti-CD133 scFv and anti-CD3ε scFv was created (Fig 1). The expression of αCD133-αCD3 engager protein was then confirmed in Lenti-X 293T cells (Fig 2). The results indicated that the αCD133-αCD3 engager could be fully translated and expressed in the mammalian cell system. In previous studies, the genetically modified T cells expressing bispecific T cell engager were generated using different approaches, including retroviral system [14–16], oncolytic adenovirus [38], and RNA electroporation [39]. In the present study, we used lentiviral vector to carry cDNA encoding αCD133-αCD3 engager to create the engineered lentiviruses, and to transduce them into healthy donor T cells [40]. The lentivirus system is safe and widely used to generate many CAR T cells, and commercially available anti-CD19 CAR T cells based on the lentivirus system have been approved by US FDA [41].

After transduction with the engineered lentiviruses, the expanded T cells were predominantly CD8^+^ cytotoxic T cells (CTLs) harboring memory and effector T cell subsets (Fig 3). Higher cytotoxic effect of CD8^+^ T cells compared to that of CD4^+^ T cells in a study of CD133-targeted bispecific antibody in glioblastoma was previously reported [23]. The memory T cell phenotype of modified T cells showed better persistence and anti-cancer effect *in vivo* [42, 43]. Thus, the increased CD8^+^ CTL population harboring the memory phenotype in our transduced T cells secreting αCD133-αCD3 engager molecules may promote increased cytotoxic activity and persistence against CCA cells *in vivo*.

Importantly, the results of the present study show that αCD133-αCD3 engager secreted from the transduced T cells could bind to both transduced and untransduced T cells (Fig 4). The binding of αCD133-αCD3 engager molecules to both transduced and untransduced (bystander) T cells could recruit both to more effectively kill CD133-expressing CCA cells. In addition, after co-culturing with CD133-expressing CCA cells, the transduced T cells secreting αCD133-αCD3 engager could proliferate and produce IFN-γ, TNF-α and granzyme B (Fig 6), which indicates that they could expand and function as designed. In a previous study, the potent anti-cancer effect of soluble CD133-targeted bispecific antibody (AC133×CD3 bsAb) was found to be mediated by CD8^+^ T cells, and it was able to recruit polyclonal T cells in glioblastoma stem cell model; however, the continuous infusion of AC133×CD3 bsAb was required [23]. Thus, it is conceivable that αCD133-αCD3 engager secreted from the transduced T cells could recruit not only the transduced T cells, but maybe also the *in situ* tumor infiltrating lymphocytes (TILs), which may resolve the problem of short lifespan of BiTE molecules and continuous infusion, and bio-distribution to the cancer tissue [44]. In the present study, the cytotoxic activities of T cells secreting αCD133-αCD3 engager were examined in both two-dimentional CCA culture and CCA spheroid culture systems, both of which demonstrated significantly higher cytotoxic activity against CD133-expressing CCA cells. A higher effector-to-target (E:T) ratio facilitated a higher level of specific cytotoxicity (Fig 5). These results confirmed the specificity of T cells secreting αCD133-αCD3 engager against CD133-expressing CCA cells, and this was observed in both a dose-dependent and antigen-dependent manner. Therefore, our newly developed T cell secreting αCD133-αCD3 engager platform has high potential for CCA treatment.

A limitation of this study is that there were no experiments in animal model. To partially mitigate this limitation, we conducted an experiment to evaluate the cytotoxic effect of T cells secreting αCD133-αCD3 engager in three-dimensional (3D) CCA spheroid system [45] in order to mimic the condition in CCA tumor mass. The results showed that, after co-culturing with the transduced T cells secreting αCD133-αCD3 engager, the CCA spheroids were markedly changed and disrupted (Fig 7). Our group previously investigated the antitumor effect of anti-CD133 CAR T cells against CCA cells, and we found CCA cell killing to be commensurate with CD133 antigen expression [27]. In this study of T cells secreting αCD133-αCD3 engager, the antitumor effect seemed to be comparable with that of anti-CD133 CAR T cells [27]. Further study to directly compare the antitumor responses of T cells secreting αCD133-αCD3 engager with those of anti-CD133 CAR T cells is warranted. In addition, despite CD133-targeted T cell-based therapy showed promising outcomes in phase II clinical trial [46], its cross-reactivity with other tissues or hematopoietic stem cells should be concerned. To ensure its safety, the T cells secreting αCD133-αCD3 engager may be modified to include suicide genes, which allow eradication of the modified T cells whenever required [47]. Further study of αCD133-αCD3 engager should be performed to confirm its safety and on-target off-tumor toxicity prior to future applications. The combination of T cells secreting αCD133-αCD3 engager with other therapeutic approaches to maximize the cytotoxic effect against CCA should also be studied to hasten the development of an optimal treatment to improve the outcomes of CCA patients.

In conclusion, our findings demonstrate proof-of-principle that T cells secreting αCD133-αCD3 engager can be an alternative approach to treating CD133-positive CCA and as well as other CD133-positive cancers, and they pave the way for future *in vivo* study and clinical trials.

## Supporting information

S1 Fig Expression and secretion of the αCD133-αCD3 engager protein. (A) Triplicate data from (B) were summarized and are shown as percentages of the engager protein normalized with GAPDH (***p<0.001). (C) Lenti-X 293T cells were transfected with pCDH.αCD133-αCD3-ENG and selected with puromycin. Intracellular and secreted αCD133-αCD3 engager proteins were examined. (A) A representative full immunoblotting of the αCD133-αCD3 engager protein in the whole cell lysates of transfected and untransfected Lenti-X 293T cells probed with anti-myc and anti-GAPDH monoclonal antibodies. (B) Triplicate data from (A) were summarized and are shown as percentages of the level of engager protein normalized with GAPDH (***p<0.001). (C) A full representative immunoblotting analysis showed the αCD133-αCD3 engager protein in the culture supernatants of the untransfected and transfected Lenti-X 293T cells.
(TIF)

S2 Fig Representative of CD133 surface expression in cholangiocarcinoma (CCA) and cholangiocytes cell lines. Cells were stained with anti-CD133 antibody under non-permeablilized condition. Flow cytometry showing surface CD133 expression (red line) versus control (black line) in KKU-100, KKU-213A CCA cells and MMNK-1 immortal cholangiocytes.
(TIF)

## References

[1] Banales JM, Cardinale V, Carpino G, Marzioni M, Andersen JB, Invernizzi P, et al. Expert consensus document: Cholangiocarcinoma: current knowledge and future perspectives consensus statement from the European Network for the Study of Cholangiocarcinoma (ENS-CCA). Nat Rev Gastroenterol Hepatol. 2016;13(5):261–80 doi: 10.1038/nrgastro.2016.51 . 27095655

[2] Banales JM, Marin JJ, Lamarca A, Rodrigues PM, Khan SA, Roberts LR, et al. Cholangiocarcinoma 2020: the next horizon in mechanisms and management. Nat Rev Gastroenterol Hepatol. 2020;17(9):557–88. doi: 10.1038/s41575-020-0310-z . 32606456 PMC7447603

[3] Rizvi S, Khan SA, Hallemeier CL, Kelley RK, Gores GJ. Cholangiocarcinoma—evolving concepts and therapeutic strategies. Nat Rev Clin Oncol. 2018;15(2):95–111. doi: 10.1038/nrclinonc.2017.157 . 28994423 PMC5819599

[4] Kokuryo T, Yokoyama Y, Nagino M. Recent advances in cancer stem cell research for cholangiocarcinoma. J Hepatobiliary Pancreat Sci. 2012;19(6):606–13. doi: 10.1007/s00534-012-0542-6 . 22907641

[5] McGrath NA, Fu J, Gu SZ, Xie C. Targeting cancer stem cells in cholangiocarcinoma (Review). Int J Oncol. 2020;57(2):397–408. doi: 10.3892/ijo.2020.5074 . 32468022 PMC7307587

[6] Wu HJ, Chu PY. Role of Cancer Stem Cells in Cholangiocarcinoma and Therapeutic Implications. Int J Mol Sci. 2019;20(17):4154. doi: 10.3390/ijms20174154 . 31450710 PMC6747544

[7] Blanco B, Compte M, Lykkemark S, Sanz L, Alvarez-Vallina L. T Cell-Redirecting Strategies to ’STAb’ Tumors: Beyond CARs and Bispecific Antibodies. Trends Immunol. 2019;40(3):243–57. doi: 10.1016/j.it.2019.01.008 . 30827461

[8] Leick MB, Maus MV, Frigault MJ. Clinical Perspective: Treatment of Aggressive B Cell Lymphomas with FDA-Approved CAR-T Cell Therapies. Mol Ther. 2021;29(2):433–41. doi: 10.1016/j.ymthe.2020.10.022 . 33130313 PMC7854294

[9] June CH, Sadelain M. Chimeric Antigen Receptor Therapy. N Engl J Med. 2018;379(1):64–73. doi: 10.1056/NEJMra1706169 . 29972754 PMC7433347

[10] Goebeler ME, Bargou RC. T cell-engaging therapies—BiTEs and beyond. Nat Rev Clin Oncol. 2020;17(7):418–34. doi: 10.1038/s41571-020-0347-5 . 32242094

[11] Tian Z, Liu M, Zhang Y, Wang X. Bispecific T cell engagers: an emerging therapy for management of hematologic malignancies. J Hematol Oncol. 2021;14(1):75. doi: 10.1186/s13045-021-01084-4 . 33941237 PMC8091790

[12] Pulte ED, Vallejo J, Przepiorka D, Nie L, Farrell AT, Goldberg KB, et al. FDA Supplemental Approval: Blinatumomab for Treatment of Relapsed and Refractory Precursor B-Cell Acute Lymphoblastic Leukemia. Oncologist. 2018;23(11): 1366–71. doi: 10.1634/theoncologist.2018-0179 . 30018129 PMC6291336

[13] Einsele H, Borghaei H, Orlowski RZ, Sublewe M, Roboz GJ, Zugmaier G, et al. The BiTE (bispecific T-cell engager) platform: Development and future potential of a targeted immuno-oncology therapy across tumor types. Cancer. 2020;126(14): 3192–201. doi: 10.1002/cncr.32909 . 32401342

[14] Iwahori K, Kakarla S, Velasquez MP, Yu F, Yi Z, Gerken C, et al. Engager T cells: a new class of antigen-specific T cells that redirect bystander T cells. Mol Ther. 2015;23(1):171–8. doi: 10.1038/mt.2014.156 . 25142939 PMC4426792

[15] Bonifant CL, Szoor A, Torres D, Joseph N, Velasquez MP, Iwahori K, et al. CD123-Engager T Cells as a Novel Immunotherapeutic for Acute Myeloid Leukemia. Mol Ther. 2016;24(9):1615–1626. doi: 10.1038/mt.2016.116 . 27401038 PMC5113097

[16] Velasquez MP, Torres D, Iwahori K, Kakarla S, Arber C, Rodriguez-Cruz T, et al. T cells expressing CD19-specific Engager Molecules for the Immunotherapy of CD19-positive Malignancies. Sci Rep. 2016;6(1): 27130. doi: 10.1038/srep27130 . 27255991 PMC4891739

[17] Mathema VB, Na-Bangchang Kesara. Current insights on cholangiocarcinoma research: a brief review. Asian Pac J Cancer Prev. 2015;16(4):1307–13. doi: 10.7314/apjcp.2015.16.4.1307 . 25743790

[18] Zhang D, Tang DG, Rycaj K. Cancer stem cells: regulation programs, immunological properties and immunotherapy. Semin Cancer Biol. 2018;52(pt 2):94–106. doi: 10.1016/j.semcancer.2018.05.001 . 29752993 PMC7859848

[19] Behrooz AB, Syahir A, Ahmad S. CD133: beyond a cancer stem cell biomarker. J Drug Target. 2019;27(3):257–69. doi: 10.1080/1061186X.2018.1479756 . 29911902

[20] Wang Y, Chen M, Wu Z, Tong C, Dai H, Guo Y, et al. CD133-directed CAR T cells for advanced metastasis malignancies: A phase I trial. Oncoimmunology. 2018;7(7):e1440169. doi: 10.1080/2162402X.2018.1440169 29900044 PMC5993480

[21] Guo Y, Feng K, Wang Y, Han W. Targeting cancer stem cells by using chimeric antigen receptor-modified T cells: a potential and curable approach for cancer treatment. Protein Cell. 2018;9(6):516–26. doi: 10.1007/s13238-017-0394-6 . 28290053 PMC5966354

[22] Zhao L, Yang Y, Zhou P, Ma H, Zhao X, He X, et al. Targeting CD133high Colorectal Cancer Cells In Vitro and In Vivo With an Asymmetric Bispecific Antibody. J Immunother. 2015;38(6):217–28. doi: 10.1097/CJI.0000000000000086 . 26049545

[23] Prasad S, Gaedicke S, Machein M, Mittler G, Braun F, Hettich M, et al. Effective Eradication of Glioblastoma Stem Cells by Local Application of an AC133/CD133-Specific T-cell-Engaging Antibody and CD8 T Cells. Cancer Res. 2015;75(11):2166–76. doi: 10.1158/0008-5472.CAN-14-2415 . 25840983

[24] Sripa B, Leungwattanawanit S, Nitta T, Wongkham C, Bhudhisawasdi V, Puapairoj A, et al. Establishment and characterization of an opisthorchiasis-associated cholangiocarcinoma cell line (KKU-100). World J Gastroenterol. 2005;11(22):3392–7. doi: 10.3748/wjg.v11.i22.3392 15948244 PMC4315993

[25] Sripa B, Seubwai W, Vaeteewoottacharn K, Sawanyawisuth K, Silsirivanit A, Kaewkong W, et al. Functional and genetic characterization of three cell lines derived from a single tumor of an Opisthorchis viverrini-associated cholangiocarcinoma patient. Hum Cell. 2020;33(3):695–708. doi: 10.1007/s13577-020-00334-w . 32207095

[26] Maruyama M, Kobayashi N, Westerman KA, Sakaguchi M, Allain JE, Totsugawa T, et al. Establishment of a highly differentiated immortalized human cholangiocyte cell line with SV40T and hTERT. Transplantation. 2004;77(3):446–51. doi: 10.1097/01.TP.0000110292.73873.25 . 14966424

[27] Sangsuwannukul T, Supimon K, Sujjitjoon J, Phanthaphol N, Chieochansin T, Phoungvarin N, et al. Anti-tumour effect of the fourth-generation chimeric antigen receptor T cells targeting CD133 against cholangiocarcinoma cells. Int Immunopharmacol. 2020;89(Pt B):107069. doi: 10.1016/j.intimp.2020.107069 . 33242709

[28] Supimon K, Sangsuwannukul T, Sujjitjoon J, Phanthaphol N, Chieochansinet T, Poungvarin N, et al. Anti-mucin 1 chimeric antigen receptor T cells for adoptive T cell therapy of cholangiocarcinoma. Sci Rep. 2021;11(1):6276. doi: 10.1038/s41598-021-85747-9 . 33737613 PMC7973425

[29] Arakawa F, Kuroki M, Kuwahara M, Senba T, Ozaki H, Matsuoka Y, et al. Cloning and sequencing of the VH and V kappa genes of an anti-CD3 monoclonal antibody, and construction of a mouse/human chimeric antibody. J Biochem. 1996;120(3):657–62. doi: 10.1093/oxfordjournals.jbchem.a021462 . 8902633

[30] Zhu X, Prasad S, Gaedicke S, Hettich M, Firat E, Niedermann G. et al. Patient-derived glioblastoma stem cells are killed by CD133-specific CAR T cells but induce the T cell aging marker CD57. Oncotarget. 2015:6(1):171–84. doi: 10.18632/oncotarget.2767 . 25426558 PMC4381586

[31] Dhatchinamoorthy K, Colbert JD, Rock KL. Cancer Immune Evasion Through Loss of MHC Class I Antigen Presentation. Front Immunol. 2021;12:636568. doi: 10.3389/fimmu.2021.636568 . 33767702 PMC7986854

[32] Maude SL, Laetsch TW, Buechner J, Rives S, Boyer M, Bittencourt H, et al. Tisagenlecleucel in Children and Young Adults with B-Cell Lymphoblastic Leukemia. N Eng J Med. 2018;378(5),439–48. doi: 10.1056/NEJMoa1709866 . 29385370 PMC5996391

[33] Hoffman LM, Gore L. Blinatumomab, a Bi-Specific Anti-CD19/CD3 BiTE(®) Antibody for the Treatment of Acute Lymphoblastic Leukemia: Perspectives and Current Pediatric Applications. Front Oncol. 2014;4:63. doi: 10.3389/fonc.2014.00063 . 24744989 PMC3978294

[34] Slaney CY, Wang P, Darcy PK, Kershaw MH. CARs versus BiTEs: A Comparison between T Cell-Redirection Strategies for Cancer Treatment. Cancer Discov. 2018;8(8)924–34. doi: 10.1158/2159-8290.CD-18-0297 . 30012854

[35] Feng KC, Guo YL, Liu Y, Dai HR, Wang Y, Lv HY, et al. Cocktail treatment with EGFR-specific and CD133-specific chimeric antigen receptor-modified T cells in a patient with advanced cholangiocarcinoma. J Hematol Oncol. 2017;10(1):4. doi: 10.1186/s13045-016-0378-7 . 28057014 PMC5217546

[36] Cai X, Li J, Yuan X, Xiao J, Dooley S, Wan X, et al. CD133 expression in cancer cells predicts poor prognosis of non-mucin producing intrahepatic cholangiocarcinoma. J Transl Med. 2018;16(1):50. doi: 10.1186/s12967-018-1423-9 . 29510695 PMC5838940

[37] Leelawat K, Thongtawee T, Narong S, Subwongcharoen S, Treepongkaruna S. Strong expression of CD133 is associated with increased cholangiocarcinoma progression. World J Gastroenterol. 2011;17(9):1192–8. doi: 10.3748/wjg.v17.i9.1192 . 21448425 PMC3063913

[38] Fajardo CA, Guedan S, Rojas LA, Moreno R, Arias-Badia M, June CH, et al. Oncolytic Adenoviral Delivery of an EGFR-Targeting T-cell Engager Improves Antitumor Efficacy. Cancer Res. 2017;77(8):2052–63. doi: 10.1158/0008-5472.CAN-16-1708 . 28143835

[39] Liu X, Barrett DM, Jiang S, Fang C, Kalos M, Grupp SA, et al. Improved anti-leukemia activities of adoptively transferred T cells expressing bispecific T-cell engager in mice. Blood Cancer J. 2016;6(6):e430. doi: 10.1038/bcj.2016.38 . 27258611 PMC5141353

[40] Milone MC, O’Doherty U. Clinical use of lentiviral vectors. Leukemia. 2018;32(7),1529–41. doi: 10.1038/s41375-018-0106-0 . 29654266 PMC6035154

[41] Labbé RP, Vessillier S, Rafiq QA. Lentiviral Vectors for T Cell Engineering: Clinical Applications, Bioprocessing and Future Perspectives. Viruses. 2021;13(8):1528. doi: 10.3390/v13081528 . 34452392 PMC8402758

[42] Busch DH, Fräßle SP, Sommermeyer D, Buchholz VR, Riddell SR. Role of memory T cell subsets for adoptive immunotherapy. Semin Immunol. 2016;28(1):28–34. doi: 10.1016/j.smim.2016.02.001 . 26976826 PMC5027130

[43] Liu Q, Sun Z, Chen L. Memory T cells: strategies for optimizing tumor immunotherapy. Protein Cell. 2020;11(8):549–64. doi: 10.1007/s13238-020-00707-9 . 32221812 PMC7381543

[44] Blanco N, Ramírez-Fernández Á, Alvarez-Vallina L. Engineering Immune Cells for in vivo Secretion of Tumor-Specific T Cell-Redirecting Bispecific Antibodies. Front Immunol. 2020;11:1792. doi: 10.3389/fimmu.2020.01792 . 32903593 PMC7438551

[45] Białkowska K, Komorowski P, Bryszewska M, Miłowska K. Spheroids as a Type of Three-Dimensional Cell Cultures-Examples of Methods of Preparation and the Most Important Application. Int J Mol Sci. 2020;21(17):6225. doi: 10.3390/ijms21176225 . 32872135 PMC7503223

[46] Dai H, Tong C, Shi D, Chen M, Guo Y, Chen D, et al. Efficacy and biomarker analysis of CD133-directed CAR T cells in advanced hepatocellular carcinoma: a single-arm, open-label, phase II trial. Oncoimmunology. 2020;9(1):1846926. doi: 10.1080/2162402X.2020.1846926 33312759 PMC7714531

[47] Jones BS, Lamb LS, Goldman F, Di Stasi A. Improving the safety of cell therapy products. Improving the safety of cell therapy products by suicide gene transfer. Front Pharmacol. 2014;5:254. doi: 10.3389/fphar.2014.00254 . 25505885 PMC4245885

