# Supplementary figures and images for: Antitumor activity of T cells secreting αCD133-αCD3 bispecific T-cell engager against cholangiocarcinoma
Source: PLoS One. 2022 Mar 21;17(3):e0265773. doi: 10.1371/journal.pone.0265773 (PMC8936442; doi:10.1371/journal.pone.0265773)

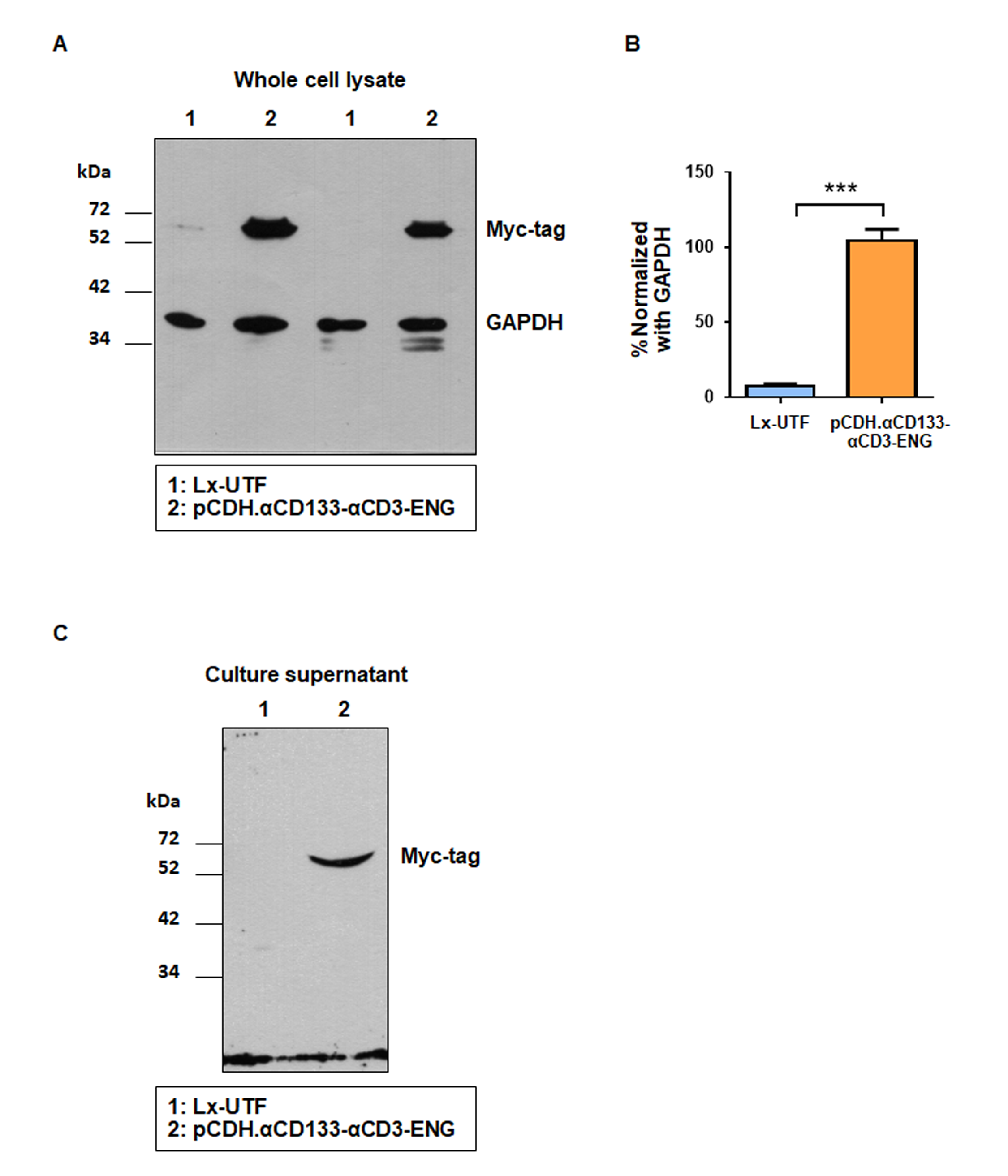

Supplement: S1 Fig — (A) Triplicate data from (B) were summarized and are shown as percentages of the engager protein normalized with GAPDH (***p<0.001). (C) Lenti-X 293T cells were transfected with pCDH.αCD133-αCD3-ENG and selected with puromycin. Intracellular and secreted αCD133-αCD3 engager proteins were examined. (A) A representative full immunoblotting of the αCD133-αCD3 engager protein in the whole cell lysates of transfected and untransfected Lenti-X 293T cells probed with anti-myc and anti-GAPDH monoclonal antibodies. (B) Triplicate data from (A) were summarized and are shown as percentages of the level of engager protein normalized with GAPDH (***p<0.001). (C) A full representative immunoblotting analysis showed the αCD133-αCD3 engager protein in the culture supernatants of the untransfected and transfected Lenti-X 293T cells. (TIF) [file pone.0265773.s001.tif]

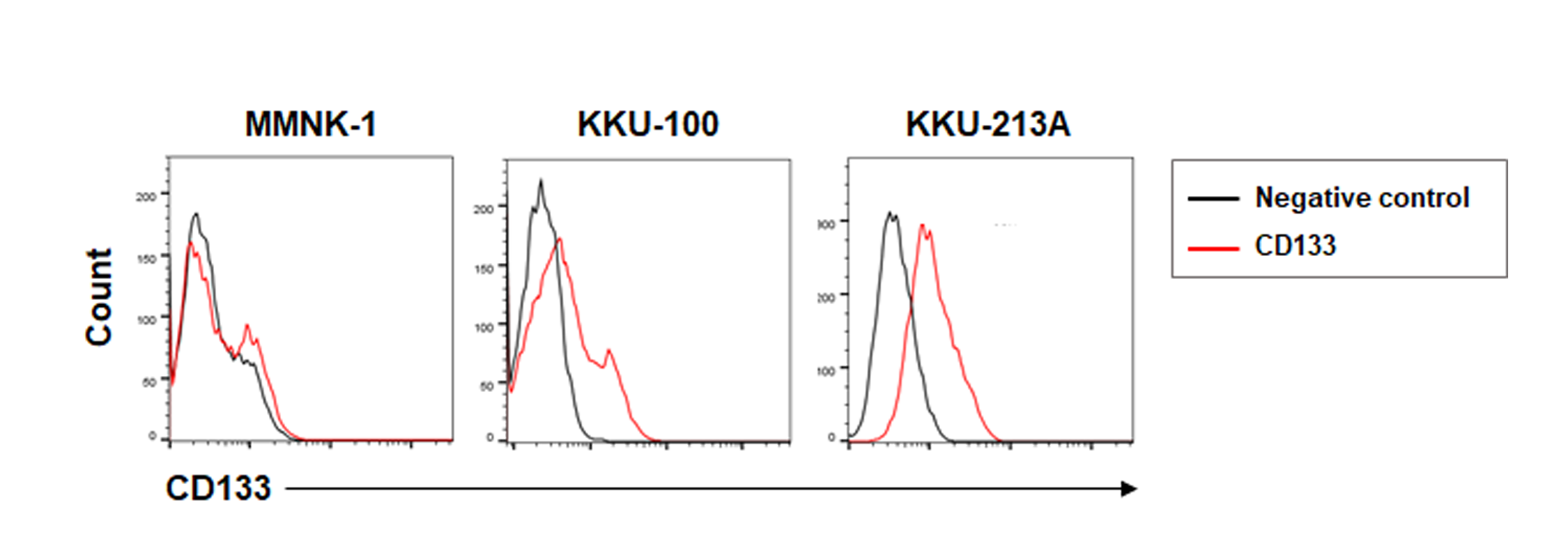

Supplement: S2 Fig — Cells were stained with anti-CD133 antibody under non-permeablilized condition. Flow cytometry showing surface CD133 expression (red line) versus control (black line) in KKU-100, KKU-213A CCA cells and MMNK-1 immortal cholangiocytes. (TIF) [file pone.0265773.s002.tif]
